# Electronic Data Capture Versus Conventional Data Collection Methods in Clinical Pain Studies: Systematic Review and Meta-Analysis

**DOI:** 10.2196/16480

**Published:** 2020-06-16

**Authors:** Lindsay A Jibb, James S Khan, Puneet Seth, Chitra Lalloo, Lauren Mulrooney, Kathryn Nicholson, Dominik A Nowak, Harneel Kaur, Alyssandra Chee-A-Tow, Joel Foster, Jennifer N Stinson

**Affiliations:** 1 Child Health Evaluative Sciences Hospital for Sick Children Toronto, ON Canada; 2 Lawrence S Bloomberg Faculty of Nursing University of Toronto Toronto, ON Canada; 3 Department of Anesthesia Mount Sinai Hospital Toronto, ON Canada; 4 Department of Anesthesia Faculty of Medicine University of Toronto Toronto, ON Canada; 5 Department of Family Medicine McMaster University Hamilton, ON Canada; 6 Institute of Health Policy, Management and Evaluation University of Toronto Toronto, ON Canada; 7 Faculty of Health Sciences University of Ottawa Ottawa, ON Canada; 8 Department of Epidemiology and Biostatistics Schulich School of Medicine and Dentistry Western University London, ON Canada; 9 Department of Family and Community Medicine Faculty of Medicine University of Toronto Toronto, ON Canada; 10 Faculty of Medicine University of Ottawa Ottawa, ON Canada; 11 Office of Education Centre for Addiction and Mental Health Toronto, ON Canada

**Keywords:** electronic, data collection, pain, efficiency, systematic review, meta-analysis

## Abstract

**Background:**

The most commonly used means to assess pain is by patient self-reported questionnaires. These questionnaires have traditionally been completed using paper-and-pencil, telephone, or in-person methods, which may limit the validity of the collected data. Electronic data capture methods represent a potential way to validly, reliably, and feasibly collect pain-related data from patients in both clinical and research settings.

**Objective:**

The aim of this study was to conduct a systematic review and meta-analysis to compare electronic and conventional pain-related data collection methods with respect to pain score equivalence, data completeness, ease of use, efficiency, and acceptability between methods.

**Methods:**

We searched the Medical Literature Analysis and Retrieval System Online (MEDLINE), Excerpta Medica Database (EMBASE), and Cochrane Central Register of Controlled Trials (CENTRAL) from database inception until November 2019. We included all peer-reviewed studies that compared electronic (any modality) and conventional (paper-, telephone-, or in-person–based) data capture methods for patient-reported pain data on one of the following outcomes: pain score equivalence, data completeness, ease of use, efficiency, and acceptability. We used random effects models to combine score equivalence data across studies that reported correlations or measures of agreement between electronic and conventional pain assessment methods.

**Results:**

A total of 53 unique studies were included in this systematic review, of which 21 were included in the meta-analysis. Overall, the pain scores reported electronically were congruent with those reported using conventional modalities, with the majority of studies (36/44, 82%) that reported on pain scores demonstrating this relationship. The weighted summary correlation coefficient of pain score equivalence from our meta-analysis was 0.92 (95% CI 0.88-0.95). Studies on data completeness, patient- or provider-reported ease of use, and efficiency generally indicated that electronic data capture methods were equivalent or superior to conventional methods. Most (19/23, 83%) studies that directly surveyed patients reported that the electronic format was the preferred data collection method.

**Conclusions:**

Electronic pain-related data capture methods are comparable with conventional methods in terms of score equivalence, data completeness, ease, efficiency, and acceptability and, if the appropriate psychometric evaluations are in place, are a feasible means to collect pain data in clinical and research settings.

## Introduction

### Background

Pain is an unpleasant sensory and emotional experience that is unique to the individual. It is also a dynamic process and fluctuates in a multidimensional manner across its sensory (eg, intensity, location, duration, etc), evaluative (ie, impact on functioning) and affective (ie, emotional effect) qualities within both the short and long term [1]. Pain is influenced by a variety of biopsychosocial factors, including genetics, mood, emotions, memory, and interpersonal relationships as well as external stimuli such as physical movement [1-3]. The accurate measurement of pain is of utmost importance to clinicians and researchers.

The most commonly used methods of measuring pain within a clinical and research context are self-reported questionnaires. Clinically, pain measurements are generally performed before and after an intervention to assess a patient’s response to therapy. These assessments are typically performed using paper-based questionnaires or via face-to-face or telephone-based verbal surveys or interviews. Although widely used, these conventional data collection methods can introduce a number of biases in the collected pain data. In particular, these methods often rely heavily on a patient’s recall of their pain symptoms (eg, pain intensity over the preceding week). Unfortunately, the recall of pain is problematic because memories of pain are vulnerable to distortion due to physical and psychological contextual factors and selective coding and retrieval of memories [4,5]. Additional issues with conventional data collection methods include limitations in conducting ecologically valid assessments of pain in the patient’s natural environment and social context, logistical challenges for repeated measurements over time, potential burden to patients, clinicians, and researchers, and possibly reduced data quality due to incomplete or back-filled pain diaries [6-8].

The advent of mobile electronic devices has created novel opportunities to collect pain-related data in clinical and research settings. Electronic data collection methods have been used to assess variables related to a variety of conditions, including mood disorders, asthma, tobacco cessation, urinary incontinence, brain injury, diabetes, cancer, and pain [7,9-11]. Specialists in pain medicine have widely advocated for the use of electronic data capture over the past two decades [12,13], and mounting evidence suggests that data collected via electronic methods may be more accurate and contain fewer errors than conventional methods [14,15]. Although randomized controlled trials and observational studies comparing electronic and conventional data collection methods suggest benefits to the use of electronic devices in pain clinical trials, no review providing an overview of these benefits currently exists. Furthermore, with the advent of smartphone-style mobile phones and their nearly ubiquitous use in developed countries [16], electronic data collection methods are becoming more widely available. As such, a review of the literature is needed to understand the potential advantages and disadvantages of collecting pain data using electronic methods.

### Objective

We aimed to identify and synthesize data from studies comparing electronic and conventional pain-related data collection methods to describe similarities and differences in pain scores, data completeness, ease of use, efficiency, and acceptability between methods.

## Methods

### Overview

We developed an internal protocol to guide the conduct of the review and meta-analysis. Reporting is guided by the Preferred Reporting Items for Systematic Reviews and Meta-Analyses [17].

### Eligibility Criteria

#### Criteria for Inclusion in the Systematic Review

To be included in this review, studies must have (1) been published in English, (2) enrolled participants in a clinical study examining an acute or chronic pain-related outcome as reported by participants, (3) used both an electronic data collection method and a conventional form of data collection (ie, paper-based, telephone, or in-person), and (4) collected data on pain score equivalence (including as part of a functional limitation or disease activity measure), data completeness, ease of use, efficiency, or acceptability between collection methods. There were no restrictions on the type of study design (randomized or observational), country of study, or year of publication. Only studies in which the full texts could be retrieved were included in the review.

#### Criteria for Inclusion in the Meta-Analysis

A subset of studies included in the systematic review was also included in the meta-analysis. These studies reported correlations or measures of agreement (ie, intraclass correlation coefficients [ICCs], Pearson correlations, Spearman rho, and weighted kappa) between patient-reported pain intensity or pain interference (including affect) scores assessed using an electronic and a conventional data capture method. Pain intensity and interference were the focus of the analysis as these constructs are commonly assessed, single-item aspects of both acute and chronic pain and are routinely used to determine treatment effectiveness and guide therapy [18,19]. As recalled pain reports may not be an accurate reflection of the momentary pain experience, we included only studies that compared momentary pain reports. No restrictions were placed on the type of data collection method (eg, mobile phone, computer-based, and tablet), pain assessment instrument (eg, numerical rating scale [NRS]), frequency of data collection, or other pain-related assessments (ie, studies that also assessed constructs such as quality of life or disease activity in addition to pain intensity or interference were included).

### Study Selection

We developed a comprehensive search strategy in consultation with a tertiary hospital librarian with expertise in the scientific literature related to digital health. We customized the search strategy to conduct tailored searches of MEDLINE, EMBASE, and Cochrane Central Register of Controlled Trials (CENTRAL) from inception until November 19, 2019. Medical Subject Headings (MeSH) keywords in the search included: *pain*, *pain measurement*, *pain threshold*, *pain perception*, *electronics*, *cellular phone*, *computers*, *handheld*, *wireless technology*, *internet*, *computer communication networks*, *mobile applications*, *randomized controlled trial*, *multicenter study*, *observational study*, *humans*, and *prospective studies*. Additional keywords used in the search included: *pain*, *pain reporting*, *personal digital assistant*, *smartphone*, and *prospective study*. An example of the search strategy can be found in Multimedia Appendix 1. We supplemented our search with searches of the author’s own databases of electronic pain assessment studies.

Search results were initially electronically screened for intradatabase and interdatabase duplicates. After the electronic removal of duplicates, titles and abstracts were screened independently by 2 authors using piloted standardized screening forms (all authors involved). Subsequently, the full texts of the included citations were reviewed in duplicate to confirm study inclusion (all authors involved). The kappa statistic was calculated as a metric of screening agreement at the full-text stage. Following the literature-based precedent, we interpreted the kappa as follows: <0.00, poor; 0.00-0.20, slight; 0.21-0.40, fair; 0.41-0.60, moderate; 0.61-0.80, substantial; and 0.81-1.00, almost perfect [20]. Disagreements among reviewers about study eligibility were resolved by consensus through discussion by at least three authors.

### Data Collection Process

A standard data collection form was created and piloted. Data abstraction occurred independently and in duplicate. Data extracted included study design, sample size, study population, electronic and conventional data collection method, duration of data collection, score equivalence between data capture methods (ie, correlations, score differences, and descriptive reports), data completeness, ease and efficiency of data collection, and patient or participant acceptability. An *a priori* decision was made to not formally assess study quality given the nature of the intervention (ie, data collection method) and the diverse study designs collected in the systematic search.

### Data Synthesis

Descriptive statistics (ie, frequencies and percentages) were used to synthesize and present data across all included studies. Meta-analysis was performed to synthesize results related to score equivalence across data capture methods. For the analysis, reported correlation coefficients (or kappa in the case of 2 studies [21,22]) served as effect size indices. In all studies where more than one coefficient for a correlation or measure of agreement between electronic and conventional pain data collection methods was available, we used the average of the coefficients so that a single study did not disproportionately impact the summary effect size. Whenever available, the reported sample size used to produce the score equivalence coefficient was used in the model. In cases where the sample size for the score equivalence analysis was not explicitly mentioned, we used the sample size reported for the entire study. Random-effects models were used to combine data across studies, and the *I^2^* statistic was used to quantify heterogeneity. The criteria set out by Higgins et al [23] were used to interpret the *I^2^* statistic; namely, 25%, 50%, and 75% were considered low, moderate, and high heterogeneity, respectively. To further examine the impact of heterogeneity on the results, the standardized residual score (ie, the standardized difference between each study effect size and the weighted mean effect size) for each study was calculated and compared [9]. A conservative cutoff of ±2 was set to examine extreme effect sizes as determined by the standardized residuals. We performed a sensitivity analysis to evaluate any impact of the type of correlation or measure of agreement on the weighted summary correlation. Specifically, following previously used methods, separate meta-analyses were conducted with studies reporting ICC or weighted kappa, which account for covariance and score mean and variability, and studies reporting the more conventional Pearson or Spearman rho coefficients [9]. Possible publication bias was assessed by visual inspection of an asymmetrical funnel plot. To investigate the sources of heterogeneity, we conducted further subgroup analyses. Our subgroup analyses focused on elucidating the impact of (1) the similarity of pain assessment measure between electronic and conventional modalities (ie, same measure or different) and (2) the duration of data collection (ie, once or multiple times). Subgroup analyses by study participant age and pain condition were precluded by the structure of data reported in our included studies. Meta-analysis procedures were conducted using Microsoft Excel (Microsoft Corporation) and Distiller SR Forest Plot Generator (Evidence Partners Inc).

## Results

### Study Selection

**Figure 1:**
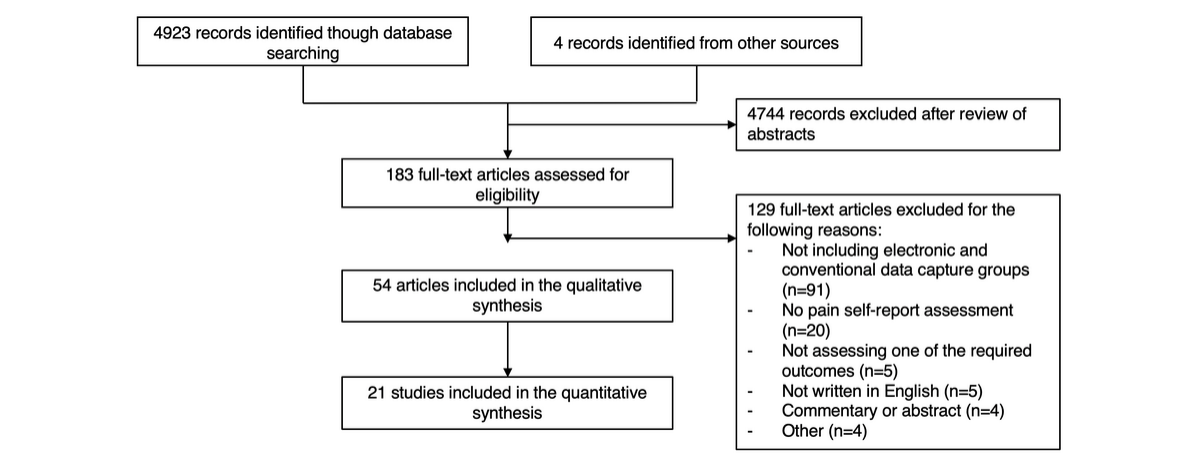
Study selection flowchart.

**Figure 2:**
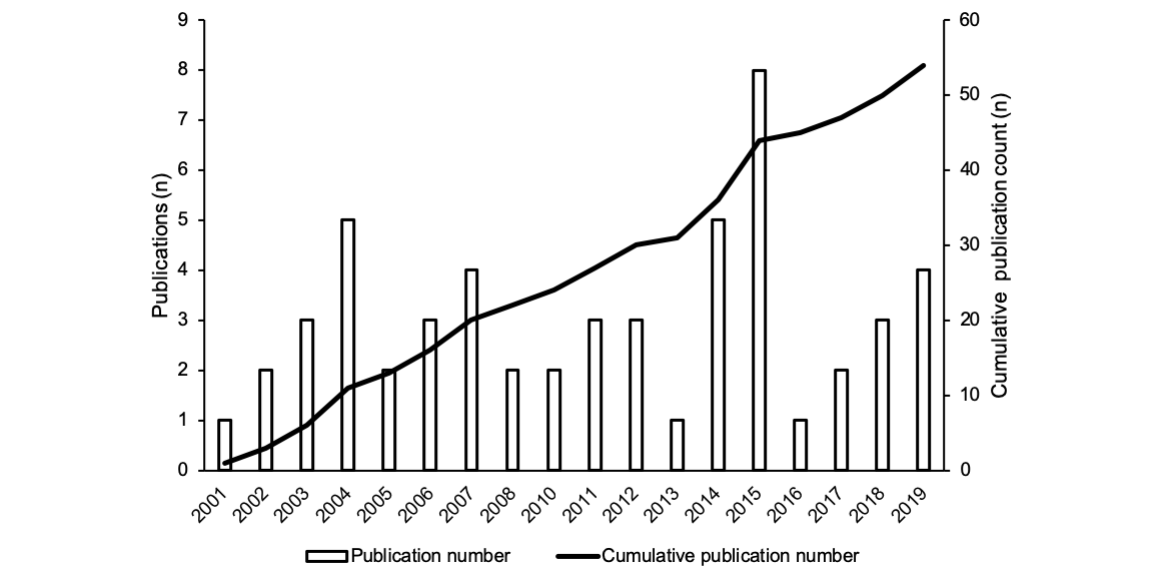
Number of studies meeting inclusion criteria overtime.

The search strategy identified 4927 studies, of which 183 underwent full-text review and 129 were excluded (Figure 1). The kappa agreement score between appraisers at this stage was 0.69, which indicated substantial agreement. In all, 54 papers reporting on 53 unique studies were included in the qualitative synthesis. Stinson et al [5,24] reported different results from the same study, so were grouped presently for analyses purposes. In all, 21 studies were included in the quantitative synthesis. The number of published studies meeting our inclusion criteria increased steadily over time (Figure 2).

### Study Characteristics

The study details are presented in Table 1. Data from a total of 7977 pain patients were included in this review. The mean number of participants across studies was 151 (range 15-2400). The average mean or median age of participants was 41.5 years (SD 17.5), and across studies, the average proportion of female participants was 63.1%; mean or median age data were missing from 9 studies and sex data were missing from 7 studies. Participants in the included studies had various painful conditions or diagnoses, including both acute and chronic pain. The most common pain conditions were nonspecific chronic pain (9/54, 17% studies), postoperative pain (8/53, 15% studies), and arthritis (8/53, 15% studies).

**Table 1:** Study characteristics.

Authors (publication year)	Criteria for electronic and conventional pain assessments	Study design	Sample size	Population (age, sex, pain condition)	Electronic data collection modality and pain data collected	Conventional data collection method and pain data collected	Duration of data collection
Allena et al (2012) [25]	Acceptability, data completeness, and ease	Not specified	85	Mean age 39.7 (SD 10.2) years, 68 females and 17 males, medication overuse headache	PDA^a^ program collecting data on pain intensity (no indication of measure), pain sensory characteristics, associated symptoms, possible trigger factors and medication use	Paper-based tool (no indication if questions were the same across formats); prospective recording of attack characteristics, more accurate descriptions	Participants completed both formats daily for 7-10 days
Athale et al (2004) [26]	Acceptability, data completeness, ease, and score equivalence	Nonrandomized, crossover	43	Mean age not specified (range 18-75+ years), 36 females and 7 males, rheumatoid arthritis	Computer program collecting data on VAS^b^-rated pain intensity, pain sensory characteristics, and affective and functional impact of pain	Paper-based tool (different from electronic format only in that pain and swelling locations are indicated on separate body maps)	Participants completed each format once
Bandarian-Balooch et al (2017) [27]	Acceptability, data completeness, ease, and score equivalence	Randomized, controlled trial	181	Mean age 26.5 (range 18-55) years, 146 female and 35 males, headache and migraine	Mobile phone or computer program collecting NRS^c^-rated pain intensity, frequency, and duration data as well as triggers and medication use	Paper-based tool with one subgroup identical to electronic format and the other a long-form report representative of conventional paper diaries	Participants completed assigned format once per day for 30 days
Bedson et al (2019) [28]	Data completeness, ease, efficiency, and score equivalence	Nonrandomized, cohort	21	Median age 62 (IQR 50-70) years, 13 females and 8 males, musculoskeletal pain	Tablet program collecting data on NRS-rated pain intensity and pain interference, as well as sleep disturbance, analgesic use, mood, and side effects	Paper-based tool (same assessment as used in the electronic study)	Participants completed electronic assessment 2 times per day for 4 weeks and the paper-based tool once at baseline and once at study completion
Bishop et al (2010) [29]	Acceptability, data completeness, ease, efficiency, and score equivalence	Randomized, crossover	167	Complete age data not reported, (range 18-78), complete sex data not reported, back pain	Computer program collecting data on the occurrence of pain interference (RMDQ^d^)	Paper-based tool (same assessment as used in the electronic format)	Participants completed each format once in random order on the same day
Blum et al (2014) [30]	Acceptability, ease, and efficiency	Crossover (randomization procedure not stated)	62	Median age 63.5 (range 23-86) years, 31 females and 31 males, cancer	PDA program (E-MOSAIC) collecting data on VAS-rated pain intensity, medication use, and other symptoms	Paper-based tool (same assessment as used in the electronic format)	Participants completed each format once with a 1-hour washout between periods
Byrom et al (2018) [31]	Score equivalence	Randomized, crossover	155	Mean age 48.6 (SD 13.1) years (range 19-69), 83 females and 72 males, chronic pain	Mobile phone or tablet program collecting data on VAS- and NRS-related pain intensity, as well as VRS^e^-rated pain intensity (SF-36^f^)	Paper-based tool (same assessment as used in the electronic format)	Participants completed each format once with a 30- to 60- min washout between periods
Castarlenas et al (2015) [22]	Acceptability, score equivalence	Crossover (randomization procedure not stated)	191	Mean age 14.6 (range 12-18) years, 117 females and 74 males, pain somewhere in their body in the last 3 months	Mobile phone program collecting data on NRS-rated pain intensity	Verbally administered tool (same assessment as used in the electronic format)	Participants completed each version once
Chiu et al (2019) [32]	Score equivalence	Randomized, crossover	138	Mean age VAS group 55 (SD 14) years, 54 females and 19 males, postoperative pain; mean age NRS group 53 (SD 13) years, 39 females and 26 males, postoperative pain	Mobile phone program collecting data on VAS- and NRS-rated pain intensity	Paper-based tool (same assessment as used in the electronic format)	Participants completed each format once with a 5-min washout between periods
Christie et al (2014) [33]	Data completeness and score equivalence	Crossover (randomization procedure not stated)	21	Median age 49.7 (SD 12.2) years, 16 females and 5 males, inflammatory rheumatic disease	Mobile phone program collecting data on NRS-rated pain intensity, fatigue, stiffness and daily activity or function	Paper-based tool (same assessment as used in the electronic format)	Participants completed each format on alternate days for 28 days
Cook et al (2004) [34]	Acceptability, ease, and score equivalence	Randomized, crossover	189	Mean age 47.5 (SD 12.8) years, 119 females and 70 males, chronic pain	Computer program collecting data on VAS- and NRS-rated pain intensity and the affective impact of pain (SF-MPQ^g^). PDI^h^ was also used.	Paper-based tool (same assessment as used in the electronic format).	Participants completed both formats once with a 45-min washout between periods
Cunha-Miranda et al (2015) [35]	Score equivalence	Nonrandomized, crossover	134	Mean age 51.3 (SD 12.0) years, 100 females and 34 males, arthritis	Tablet program collecting data on VAS-rated pain intensity and interference, as well as other disease and quality of life metrics dependent on participant diagnosis	Paper-based tool (same assessment as used in the electronic format).	Participants completed each format with a 15-min washout between periods
Fanciullo et al (2007) [36]	Acceptability and score equivalence	Crossover (randomization procedure not stated)	54	Median age 10.7 (SD 4.0) years, 26 females and 28 males, various causes of pain (eg, broken bones, infections, and cancer)	Computer program collecting data on pain intensity from an investigator-developed computer faces scale	Paper-based tool (Wong-Baker Faces Scale)	Participants completed both formats once
Freynhagen et al (2006) [37]	Ease	Nonrandomized, cohort	717	Mean age 56.0 years (SD not stated), sex ratio not specified, chronic pain	PDA program collecting data on VAS-rated pain intensity, functional disability, and depression	Paper-based tool (same assessment as used in the electronic format)	Participants completed either format once
Gaertner et al (2004) [38]	Acceptability, data completeness, ease, efficiency, and score equivalence	Randomized, crossover	24	Mean age 49.9 (SD 15.1) years, 13 females and 11 males, various painful conditions (eg, cancer, osteoarthritis, chronic neuropathic pain)	PDA program collecting data on NRS-rated pain intensity, analgesic use, other symptoms and therapies	Paper-based tool (same assessment as used in the electronic format)	Participants completed each format daily for 14 days
Garcia-Palacios et al (2013) [39]	Acceptability, data completeness, ease, and score equivalence	Randomized, crossover	47	Mean age 48.1 (SD 8.0) years, 47 females, fibromyalgia	Mobile phone program collecting data on NRS-rated pain intensity, fatigue, and faces scale-rated mood. BPI^i^ and fatigue scale were also used.	Paper-based tool (same assessment as used in the electronic format)	Participants completed the electronic assessment 3 times per day for 1 week and the paper-based tool once per week
Heiberg et al (2007) [40]	Acceptability, data completeness, efficiency, and score equivalence	Crossover (randomization procedure not stated)	38	Mean age 58.4 (SD 12.9) years, 25 females and 12 males, rheumatoid arthritis	PDA program collecting data on VAS-rated pain intensity, fatigue, and global disease activity, as well as NRS-rated pain intensity (RADAI^j^) daily, and VRS-rated pain intensity and interference (SF-36) and additional questions on daily functioning collected weekly	Paper-based tool (same assessment as used in the electronic format)	Participants completed each format for 42 days or 6 weeks (21 days/3 weeks for each format)
Hofstedt et al (2019) [41]	Acceptability and score equivalence	Nonrandomized, cohort	70	Mean age 51.7 (SD 13.2) years, 53 females and 17 males, arthritis	Computer, tablet, or mobile phone program collecting data on VAS-rated pain intensity, global health, and fatigue, as well as disease activity and functional index for a subset of patients	Paper-based tool (same assessment as used in the electronic format)	Participants completed the electronic format at least once during the week before a clinic appointment and the conventional format once at the appointment
Jaatun et al (2014) [42]	Acceptability, ease, score equivalence	Randomized, crossover	92	Age range 20-90 years, 33 females and 59 males, cancer	Tablet program collecting data on pain location from an investigator-developed pain map	Paper-based tool collecting pain location data from the BPI	Participants completed both formats once a 20-30-min washout between periods
Jamison et al (2001) [15]	Data completeness and score equivalence	Nonrandomized, cohort	36	Mean age 42.6 (SD 7.0) years, 20 females and 16 males, chronic low back pain	PDA program collecting data on VAS-rated pain intensity each hour for 16 waking hours as well as number of sleep hours	Paper-based tool collecting data on NRS-rated pain intensity for each waking hour and telephone-based NRS-pain intensity over the preceding week	Participants completed formats for 1 year.
Jamison et al (2002) [43]	Score equivalence	Randomized, crossover	24	Mean age 34.4 (range 19-57) years, 19 females and 5 males, healthy volunteers holding weights heavy enough to induce pain	PDA program collecting data on VAS-rated pain intensity	Paper-based tool (same assessment as used in the electronic format)	Participants completed each format 21 times on 1 day
Jamison et al (2006) [44]	Score equivalence	Nonrandomized, cohort	21	Mean age 42.0 (SD 4.9) years, 9 females and 12 males, low back pain	PDA program collecting data on VAS-rated pain intensity, as well as the affective and functional impact of pain, medications, and side effects	Telephone interviews collecting data on recalled NRS-rated pain over the previous week and telephone-based NRS-pain intensity over the preceding week	Participants completed the electronic format at least daily for 1 year.
Jonassaint et al (2015) [45]	Score equivalence	Nonrandomized, cohort	15	Median age 29 (range 16-54) years, 6 females and 9 males, sickle cell disease	Mobile phone program collecting VAS-rated pain intensity, location and perceived severity, and treatment strategies.	Paper-based tool collecting data on VAS-rated pain (same assessment as used in the electronic format)	Participants first completed paper-based tool, then electronic version daily for 28 days.
Junker et al (2008) [46]	Data completeness and score equivalence	Randomized, crossover	198	Mean age 56.5 (SD 13.9) years, 114 females and 84 males, chronic pain	PDA program collecting data on VAS-rated pain intensity recalled pain over previous 4 weeks, recalled worst pain in previous 4 weeks and a summative pain score	Paper-based tool (different from electronic format in that pain intensity rated on NRS)	Participants completed each format once
Khan et al (2019) [47]	Acceptability and data completeness	Randomized, cohort	78	Mean age 52.7 (SD 11.1) years, 78 females, postoperative pain	Computer, mobile phone, or tablet program collecting data on data on NRS-related pain intensity, as well as pain catastrophizing, preoperative anxiety, and somatic preoccupation presurgery and medication use and adverse events postsurgery	Paper- or in-person verbal tool (same assessment as used in the electronic format)	Participants completed each format twice daily on postoperative days 1, 2, 3, and 9 and at a 3-month follow-up visit
Kim et al (2016) [48]	Acceptability and efficiency	Nonrandomized, cohort	96	Mean age not specified, 59 females and 37 males, spinal disorders	Tablet program collecting data on VAS-rated pain intensity, disability, as well as questions related to the nature of pain and alleviating and aggravating pain factors	Paper-based tool (same assessment as used in electronic format)	Each format used for a variable and unspecified number of times
Koho et al (2014) [49]	Acceptability, ease, and score equivalence	Randomized, crossover	94	Mean age 47.0 (SD 8.0) years, 55 females and 39 males, chronic musculoskeletal pain	Computer program collecting data on the affective impact of pain	Paper-based tool (same assessment as used in the electronic format)	Participants completed each format twice on two consecutive days
Kvien et al 2005 [50]	Acceptability, efficiency, and score equivalence	Nonrandomized, crossover	30	Mean age 61.6 (range 49.8-70.0) years, 19 females and 11 males, rheumatoid arthritis	PDA program collecting data on VAS-rated pain intensity, fatigue, and patient global evaluation of their disease, NRS-rated pain intensity (RADAI), VRS-rated pain intensity and interference (SF-36), and additional questions on daily functioning	Paper-based tool (same assessment as used in the electronic format)	Participants completed each format on 2 occasions 5 to 7 days apart
MacKenzie et al (2011) [51]	Acceptability, ease, efficiency, and score equivalence	Randomized, crossover	63	Mean age 53.0 (range 28.0-82.0) years, 29 females and 34 males, psoriatic arthritis	Computer program collecting data on VAS-rated pain intensity (HAQ^k^), VRS-rated pain intensity and interference (SF-36) and additional questions on health and arthritis-related symptoms and function	Paper-based tool (same assessment as used in the electronic format)	Participants completed each format once 1 hour apart
Marceau et al (2007) [52]	Acceptability, data completeness, ease and score equivalence	Randomized, crossover	36	Mean age 48.0 (SD 8.0) years, 25 females and 11 males, chronic pain	PDA program collecting data on VAS-rated pain intensity and interference, as well as on the affective impact of pain, medication use, and pain location	Paper-based tool (same assessment as used in the electronic format)	Participants completed each format once per day for 2 weeks with a 1-week washout between periods
Marceau et al (2010) [53]	Acceptability and ease	Randomized, controlled trial	134	Mean age 49.5 (SD 11.3) years, 67 females and 67 males, chronic pain	PDA program collecting data on VAS-rated pain intensity and interference, as well as on the affective impact of pain, medication use, and pain location	Paper-based tool (same assessment as used in the electronic format)	Participants completed each format monthly for 10 months
Matthews et al (2018) [54]	Score equivalence	Randomized, crossover	32	Mean age 24.5 (SD 5.6) years, 25 females and 7 males, nontraumatic knee pain	Tablet-based method of collecting data on pain area, location, and distribution through drawing	Paper-based tool (same assessment as used in the electronic format)	Participants completed each format once with a 1-2-min washout between periods
Neudecker et al (2006) [55]	Score equivalence	Randomized, crossover	53	Mean age 51.0 (range 18.0-78.0) years, 33 females and 20 males, postoperative pain	PDA program collecting data on VAS-rated pain intensity	Manually manipulated slide device-based tool (same assessment as used in the electronic format)	Participants completed each format while participants were at rest and while coughing (number of assessments not specified)
Palermo et al (2004) [56]	Acceptability, data completeness, ease, and score equivalence	Randomized, controlled trial	60	Mean age electronic version 12.3 (SD 2.4) years, mean age paper version 12.3 (SD 3.0) years, 42 females and 18 males, headache or juvenile idiopathic arthritis	PDA program collecting data on faces scale-rated pain intensity, pain sensory characteristics, affective and functional impact of pain	Paper-based tool (same assessment as used in the electronic format)	Participants completed the assigned format for 7 consecutive days
Pawar et al (2017) [57]	Acceptability, ease, efficiency, and score equivalence	Randomized, crossover	52	Mean age 46.6 (SD 14.5) years, 31 females and 21 males, low back pain	Mobile phone program collecting data on the occurrence of pain interference (RMDQ)	Paper-based tool (same assessment as used in the electronic format)	Participants completed each format with a 1-hour interval between assessments
Ritter et al (2004) [58]	Data completeness and score equivalence	Randomized, controlled trial	397	Mean age electronic version 45.9 (SD 14.3) years, mean age paper version 44.6 (SD 13.5) years, 287 females and 110 males, diabetes, asthma, heart disease, lung disease, hypertension	Computer program collecting data on 16 health-related variables including NRS-rated pain intensity	Paper-based tool (same assessment as used in the electronic format)	Participants completed assigned format once
Rolfson et al (2011) [59]	Data completeness and score equivalence	Randomized, controlled trial	2400	Group mean age and sex ratio not specified, total hip replacement surgical pain	Computer program collecting data on VAS-rated pain intensity and health-related quality of life	Paper-based tool (same assessment as used in the electronic format)	Participants completed assigned format once
Saleh et al (2002) [60]	Acceptability and score equivalence	Nonrandomized, cohort	87	Mean age 63.5 (SD 11.6) years, 3 females and 84 males, hip or knee pain	PDA program collecting data on VRS-rated pain intensity and interference (SF-36) and NRS-rated pain interference (WOMAC^l^)	Paper-based tool (same assessment as used in the electronic format)	Participants completed assigned format once
Sanchez-Rodrıguez et al (2015) [61]	Acceptability and score equivalence	Nonrandomized, crossover	180	Mean age 14.9 (SD 1.64; age range: 12–19) years, 104 females and 76 males, pain in the last 3 months	Mobile phone program, collecting NRS-, faces pain scale-, VAS-and CAS^m^-pain intensity data	Paper-based tool (same assessment as used in the electronic format)	Participants completed each assigned format once with a 30-min interval between assessments
Serif et al 2005 [62]	Ease and efficiency	Nonrandomized, cohort	50	Age range 27-65 years, sex not specified, back pain	PDA program collecting data on VAS-pain intensity, pain location, and other symptoms	Paper-based tool (same assessment as used in the electronic format)	Participants completed assessments every 2 hours (between 10 am and 4 pm) for 5 days
Stinson et al (2008 and 2014) [5,24]	Acceptability, data completeness, ease, efficiency, and score equivalence	Nonrandomized, cohort	76 in nonjoint injection group and 36 in joint injection group	Mean age nonjoint injection group 13.4 (SD 2.5) years, 59 females and 17 males, arthritis; mean age joint injection group 12.6 (SD 2.4) years, 24 females and 12 males, arthritis	PDA program collecting data on VAS-rated pain intensity, interference and unpleasantness	Paper based tool (different from the electronic tool in that recall period was 1 week) and quality of life and pain coping also assessed	Participants completed the electronic format 3 times daily for 14 days (21 days for joint injection group) and the conventional format on days 7 and 14 (and 21 for joint injection group)
Stinson et al (2012) [63]	Acceptability, data completeness, ease, efficiency, and score equivalence	Randomized, crossover	24 children aged 4-7 years (with parents) and 77 youth aged 8-18 years	Mean age younger children 5.9 (SD 0.9) years, mean age older children 13.5 (SD 3.1) years, 61 females and 36 males, various rheumatic diseases	(1) Mobile phone program collecting data on faces scale or NRS-rated pain intensity, pain sensory characteristics and affective and functional impact of pain and (2) computer program (same assessment as used in the mobile phone format)	Paper-based tool (same assessment as used in the electronic formats)	Participants completed each format once
Stinson et al (2015) [7]	Acceptability, data completeness, ease, efficiency, and score equivalence	Nonrandomized, cohort	92 in nonsurgical group and 14 in surgical group	Mean age nonsurgical group 13.1 (SD 2.9) years, 45 females and 47 males, cancer; mean age surgical group 14.8 (SD 2.8) years, 7 females and 7 males, cancer surgery	Mobile phone program collecting data on VAS-rated pain intensity, interference and unpleasantness, as well as pain duration and location, pain management strategies used	Paper-based tool (different from the electronic tool in that recall period was 1 week) and quality of life and pain coping also assessed	Participants completed the electronic format twice daily for 14 days (21 days for surgical group) and the conventional format on days 7 and 14 (and 21 for surgical group)
Stomberg et al (2012) [64]	Acceptability, data completeness, ease, efficiency, and score equivalence	Randomized, controlled trial	40	Age range 18-66 years, sex ratio not specified, posthysterectomy and postcholecystectomy pain	Mobile phone program collecting data on NRS-rated pain intensity	Paper-based tool (same assessment as used in the electronic format)	Participants in the electronic group completed pain assessments every 4 hours during the day for 6 days, plus ad hoc reports, participants in the conventional group completed pain assessments every 4 hours during the day for 4 days
Stone et al (2003) [65]	Data completeness and score equivalence	Randomized, controlled trial	91	Mean age across groups 49.0-53.5 (SD 10.4-10.7) years, 77 females and 14 males, chronic pain	PDA program collecting data on VAS-rated pain intensity, pain sensory characteristics, and affective and functional impact of pain	Paper-based tool (same assessment as used in the electronic format)	Participants in the electronic group completed pain assessments either 3, 6, or 12 times per day for 2 weeks, participants in the conventional group completed pain assessments once per week for 2 weeks.
Sun et al (2015) [66]	Acceptability and score equivalence	Randomized, crossover	128	Median age faces pain scale group 7.5 (range 4-12 years), median age CAS group 13 (range 5-18 years), 52 females and 76 males, postoperative pain	Mobile phone program collecting data on faces pain scale- (children <5 years) and CAS- (children 5-12 years) rated pain intensity	Paper-based tool (same assessment as used in the electronic format)	Participants completed each tool within 10 min of waking from surgery and 30 min later with a 5-min washout interval in between
Suso-Ribera et al (2018) [67]	Data completeness, ease, and score equivalence	Nonrandomized, cohort	38	Mean age 42.7 (SD 9.9) years, 20 females and 18 males, chronic pain	Mobile phone-based program collecting data on NRS-rated pain intensity and interference, as well as pain catastrophizing, pain acceptance, and fear and avoidance, mood and coping	Paper- and telephone-based tool collecting data on NRS-rated pain intensity and interference, as well as pain catastrophizing, pain acceptance, and fear/avoidance, mood and coping (tools used may have differed from electronic format)	Participants completed the electronic format twice daily for 30 days and the conventional format at baseline and after each study week
Symonds et al (2015) [68]	Score equivalence	Nonrandomized, crossover	356	Mean age across groups 58.4 (SD 8.4) years, 279 females and 77 males, osteoarthritis of the index knee	PDA program collecting data on VRS-rated pain intensity and interference (SF-36) and NRS-rated pain interference (WOMAC)	Paper-based tool collected data from the WOMAC	Participants complete each format once (washout period not specified)
Theiler et al (2007) [69]	Acceptability	Nonrandomized, cohort	60	Mean age 52.1 (range 23.0-79.0) years, 36 females and 24 males, chronic pain	Computer program collecting data on NRS-rated pain intensity, medication use, and other symptoms	Telephone-based tool (same assessment as used in the electronic format)	Participants completed either format every day for 1 week followed by 3-4 days per week for 3 additional weeks
VanDenKerkhof et al (2003) [70]	Data completeness, efficiency, and score equivalence	Nonrandomized, cohort	84	Age and sex ratio not specified, postorthopedic surgical pain	PDA-based program collecting data on NRS-rated pain intensity and physician orders	Paper-based tool (same assessment as used in the electronic format)	Physician completed each format for half of the study period, assessments were completed once per participant
VanDenKerkhof et al (2004) [71]	Data completeness and efficiency	Randomized, controlled trial	74	Mean age electronic group 64.0 (SD 10.0) years, mean age conventional group 58.0 (SD 16.0) years, sex ratio not specified, postorthopedic surgical pain	PDA program collecting data on NRS-rated pain intensity and physician orders	Paper-based tool (same assessment as used in the electronic format)	Participants completed assigned format once
Wæhrens et al (2015) [72]	Acceptability, ease, and score equivalence	Randomized, crossover	20	Mean age 47.8 (SD 11.0) years, 20 females, chronic widespread pain	Computer program collecting data on NRS-rated pain intensity, interference, affect as part of the FIQ^n^, as well as measures of depression, quality of life, coping and anxiety	Paper based tool (same assessment as used in the electronic format)	Participants completed each format once with a 5-min wash-out interval
Wood et al (2011) [21]	Acceptability and score equivalence	Randomized, crossover	202	Mean age 8.3 (SD 2.6) years, 85 females and 117 males, postoperative or disease-related pain	PDA program collecting data on faces scale-rated pain intensity	Paper-based tool (same assessment as used in the electronic format)	Participants completed each format once with a 30-min washout between periods

^a^PDA: personal digital assistant.

^b^VAS: Visual Analog Scale.

^c^NRS: Numerical Rating Scale.

^d^RMDQ: Roland Morris Disability Questionnaire.

^e^VRS: Verbal Rating Scale.

^f^SF-36: Short Form 36 Health Survey.

^g^SF-MPQ: Short Form McGill Pain Questionnaire.

^h^PDI: Pain Disability Index.

^i^BPI: Brief Pain Inventory.

^j^RADAI: Rheumatoid Arthritis Disease Activity Index.

^k^HAQ: Health Assessment Questionnaire.

^l^WOMAC: Western Ontario and McMaster University Osteoarthritis Index.

^m^CAS: Color Analogue Scale.

^n^FIQ: Fibromyalgia Impact Questionnaire.

Regarding electronic data capture modalities, the devices used for data collection included the following: personal digital assistants (PDA; 22/53, 41%), computer (either Web-based or offline; 10/53, 18%), smartphone (9/53, 17%), tablet (5/53, 9%), mobile phones, tablets, and//or computers (6/53, 11%), and conventional mobile phone (1/53, 22%). Studies conducted more recently tended to use non-PDA–based mobile modalities, whereas older studies utilized PDA and computer-based modalities of assessment (average year of publication for studies employing non-PDA mobile devices was 2016 versus 2007 for studies on PDA and computer-based modalities). Conventional pain assessment modalities were paper-based (46/53, 86.7%), telephone-interviews (2/53; 43%), paper- and verbal-based (3/53, 65%), face-to-face interviews (1/53, 22%), and a manually manipulated slide device (1/53, 22%).

In total, 35% (19/53) studies used a randomized, crossover design, 14 (26%) studies used a nonrandomized cohort design, 9 (17%) studies were randomized controlled trials, 5 (9%)studies used a nonrandomized crossover design, 5 (9%) studies used a crossover design with unclear randomization (no mention of whether a randomization procedure was employed), and 1 (22%) study did not specify the study design. The duration of data collection varied across studies, ranging from a single assessment being conducted to repeated assessments over the course of a year.

### Data Related to Pain Assessment Measures

Pain intensity was the most commonly assessed pain outcome, measured in 90% (48/53) of studies. Methods to measure pain intensity using electronic methods were visual analog scales (VAS; 26/53, 49%), NRS (22/53, 41%), faces scales (5/53, 9%), verbal rating scales (5/53, 9%), and color analogue scales (2/53, 44%). The method of pain intensity measurement was not specified in 1 study (21.9%). In total, 75% (40/53) of studies employed the same measurement tools across the electronic and conventional modalities.

Pain assessment tools using electronic data capture most often were multidimensional in nature (35/53, 66%). Electronic data collection methods were used to capture multidimensional aspects of pain using the following validated questionnaires: Brief Pain Inventory, Fibromyalgia Impact Questionnaire, Health Assessment Questionnaire, Pain Disability Index, Rheumatoid Arthritis Disease Activity Index, Roland-Morris Disability Questionnaire, Short Form 20, Short Form 36, Short Form McGill Pain Questionnaire, and Western Ontario and McMaster Universities Arthritis Index.

### Comparisons Across Data Collection Modalities

#### Qualitative Synthesis of Score Equivalence

In total, 83% (44/53) of studies reported pain score equivalence between electronic and conventional data capture methods (Table 2). Statistical methods used to compare scores differed between studies: 47% (21/44) of these studies used correlational analyses (ie, ICC, Pearson coefficient, Spearman coefficient, or weighted kappa) to examine the agreement between pain scores; 29% (13/44) studies statistically examined the differences between mean or median score, SDs, or ranges between methods; 76% (3/44) studies used descriptive methods to examine agreement; and 15% (7/44) studies used a combination of these statistical methods.

**Table 2:** Summary of study results related to score equivalence.

Outcome and study (year)		Equivalence examination method and results				
		Score correlation		Score differences		Descriptive
		Method	Results	Method	Results	
**Studies reporting pain score equivalence**						
	Athale et al (2004) [26]	ICC^a^	Pain intensity ICC=0.941; pain interference ICC=0.959	—^b^	—	—
	Bandarian-Balooch et al (2017) [27]	—	—	ANOVA^c^	Mean pain intensity, frequency, duration, medication usage, disability *P*>.05 of all	—
	Bishop et al (2010) [29]	ICC	Pain interference ICC=0.965	—	—	Mean low-back pain interference score difference between method 0.03 (SD 1.43; 95% CI −0.19 to 0.25). Authors predefined acceptable 95% CI was ± 0.5.
	Byrom et al (2018) [31]	ICC	Pain intensity *r*=0.87-0.98, 95% CI 0.83-0.99)	—	—	—
	Castarlanas et al (2015) [22]	Weighted kappa	Pain intensity κ=0.813	—	—	—
	Chiu et al (2019) [32]	Pearson correlation	Pain intensity *r*=0.93-0.96 (*P*<.001)	—	—	Using Bland-Altman method, an agreement between the data capture techniques shown at 95% CI.
	Christie et al (2014) [33]	—	—	Paired sample *t* tests or Wilcoxon Signed Rank Test	Mean, SD, and range of pain intensity *P*>.46 for all	—
	Cook et al (2004) [34]	Spearman rho	Pain intensity and interference rho=0.67-084	—	—	—
	Cunha Miranda et al (2015) [35]	ICC	Pain intensity and interference ICC=>0.781-0.944	—	—	—
	Fanciullo et al (2007) [36]	Spearman rho	Pain intensity rho=−0.72 (*P*<.001)	—	—	—
	Gaertner et al (2004) [38]	—	—	*t* test	Mean pain intensity not significantly different (*P* value not reported)	—
	Garcia-Palacios et al (2013) [39]	Pearson correlation	Pain intensity *r*=0.79 (*P*<.001)	—	—	—
	Heiberg et al (2007) [40]	—	—	Wilcoxon’s signed rank test	Mean, SD, and range of pain intensity *P*>.06	—
	Hofstedt et al (2019) [41]	ICC	Pain intensity ICC=0.952	Paired *t* test	Mean pain intensity not significantly different (*P*=.29)	Using Bland-Altman method, an agreement between the data capture techniques shown at 95% CI.
	Jaatun et al (2014) [42]	—	—	—	—	In 71% (65/92) of cases participants marked the same number of areas and the same anatomical locations on both body map versions, in 20 cases, the markings were relatively similar, and in 7 cases, the markings were dissimilar.
	Jamison et al (2001) [15]	Pearson correlation	Pain intensity *r*=0.88, *P*<.001	—	—	—
	Jamison et al (2002) [43]	Pearson correlation	Pain intensity *r*^2^>0.999	—	—	—
	Jamison et al (2006) [44]	Pearson correlation	Pain intensity *r*=0.99 (95% CI 0.975-0.996)	—	—	—
	Jonassaint et al (2015) [45]	ICC	Pain intensity ICC=0.97 (95% CI 0.88-0.99)	—	—	—
	Kvien et al (2005) [50]	Pearson correlation	Pain intensity *r*=0.79-0.93	—	—	—
	MacKenzie et al (2011) [51]	ICC	Pain intensity and interference ICC=0.95-0.97; 95% CI 0.95-0.98)	—	—	—
	Marceau et al (2007) [52]	—	—	—	—	Participants reported similar using each data capture methods for pain intensity, pain interference, mood, and helpfulness of medications.
	Matthews et al (2018) [54]	Pearson correlation and ICC	Pain location pixelated area *r*=0.93 (*P*<.001) and ICC=0.966 (*P*<.001)	*t* test	Mean pain location pixelated area not significantly different (*P*=.93)	Using Bland-Altman method, an agreement between the data capture techniques shown at 95% CI.
	Neudecker et al (2006) [55]	Pearson correlation	Pain intensity *r*=0.902 (*P*<.001)	—	—	—
	Palermo et al (2004) [56]	—	—	*t* test	Mean pain intensity not significantly different (*P* value not reported)	—
	Pawar et al (2017) [57]	ICC	Pain interference ICC=0.994 (95% CI 0.989-0.996)	—	—	—
	Ritter et al (2004) [58]	—	—	*t* test, Wilcoxon’s signed rank test and ANCOVA^d^	Mean pain intensity and pain interference *P*>.30	—
	Saleh et al (2002) [60]	—	—	Test not reported	Mean and SD pain intensity and interference not significantly different (*P* value not reported)	—
	Sanchez-Rodrıguez et al (2015) [61]	—	—	—	—	Using Bland-Altman method, an agreement between the data capture techniques shown for the FPS-R^e^, the VAS^f^, and the CAS^g^ at 95% CI. Agreement for the NRS^h^-11 shown in the 80% CI level.
	Stinson et al (2012) [63]	—	—	*t* test	Mean pain intensity *P*>.09 for younger and older children	—
	Stinson et al (2015) [7]	Pearson correlation	Pain intensity *r*=0.49-0.63 (*P*<.001); pain interference *r*=0.53-0.65 (*P*<.001)	—	—	—
	Stone et al (2003) [65]	—	—	Repeated-measures ANOVA	Mean pain intensity *P*>.16	—
	Sun et al (2015) [66]	Pearson correlation	Pain intensity *r*=0.87-0.93	—	—	Using Bland-Altman method, agreement between the data capture techniques shown in the 80% CI level.
	Symonds et al (2015) [68]	Pearson correlation and ICC	Pain intensity *r*=0.92 and ICC=0.92; pain interference *r*=0.97 and ICC=0.97	—	—	—
	VanDenKerkhof et al (2003) [70]	—	—	Mann-Whitney test	Median pain intensity not significantly different (*P* value not reported)	—
	Wood et al (2011) [21]	Weighted kappa and Spearman rho	Pain intensity κ 0.846 (95% CI 0.79-0.896) and rho=0.911 (*P*<.001)	—	—	—
**Studies reporting pain score nonequivalence**						
	Rolfson et al (2011) [59]	—	—	Mann-Whitney *U* test	Mean pain intensity *P*=.02	—
**Studies reporting discrepant results**						
	Bedson et al (2019) [28]	Spearman rho	Pain intensity and interference baseline paper-based and first 3 days of electronic reports rho=0.60 −0.79 (*P*<.006); pain intensity and interference last 3 days of electronic reports and follow-up paper-based rho=0.40 (*P*<.11)-0.92 (*P*<.001)	—	—	—
	Junker et al (2008) [46]	—	—	Paired *t* test	Mean average and present pain intensity *P*<.01; mean worst pain *P*=.68 (null hypothesis was nonequivalence)	—
	Koho et al (2014) [49]	ICC	Pain-related fear ICC=0.77 (95% CI 0.66-0.85)	Test not reported	Significantly higher mean scores for 2 of 17 scale items using the electronic method (*P* value not reported)	Using Bland-Altman method, an agreement between the data capture techniques shown at 95% CI.
	Stinson et al (2008 and 2014) [5,24]	Pearson correlation and ICC	Pain intensity *r*=0.55-0.76 and ICC=0.52-0.75 (*P*<.01); pain interference *r*=0.77-0.84 (*P*<.01)	—	—	—
	Stomberg et al (2012) [64]	—	—	Mantel’s test	Mean pain intensity significantly higher in electronic data capture group on 2 of 3 assessment days (*P* value not reported)	—
	Suso-Ribera et al (2018) [67]	Pearson correlation	Pain intensity and interference *r*=0.60-0.81	Paired sample *t* tests	Averaged weekly pain interference reports from app significantly lower than verbally or paper-based recalled interference verbal over the week *P*<.001	—
	Wæhrens et al (2015) [72]	ICC	Pain intensity and pain interference ICC=0.76-0.98 (95% CI 0.50-0.99)	—	—	—

^a^ICC: intraclass correlation coefficient.

^b^N/A: not applicable.

^c^ANOVA: analysis of variance.

^d^ANCOVA: analysis of covariance.

^e^FPS-R: Faces Pain Scale-Revised

^f^VAS: Visual Analog Scale.

^g^CAS: Color Analogue Scale.

^h^NRS: Numerical Rating Scale.

Across all methods used to compare scores, 82% (36/44) studies demonstrated equivalence between scores reported electronically or using conventional methods. One of these 44 studies (2%) reported nonequivalent scores between data collection methods, and 16% (7/44) studies reported discrepant results. Among studies reporting nonequivalence or discrepancies, purported reasons were recall bias, differences in question layout wherein paper assessments made all items visible to participants simultaneously allowing item scoring in relation to other responses, capacity to change item response using paper methods, and differences in scale presentation (eg, numerical values for NRS not shown using electronic data capture method).

#### Quantitative Synthesis of Score Equivalence

**Figure 3:**
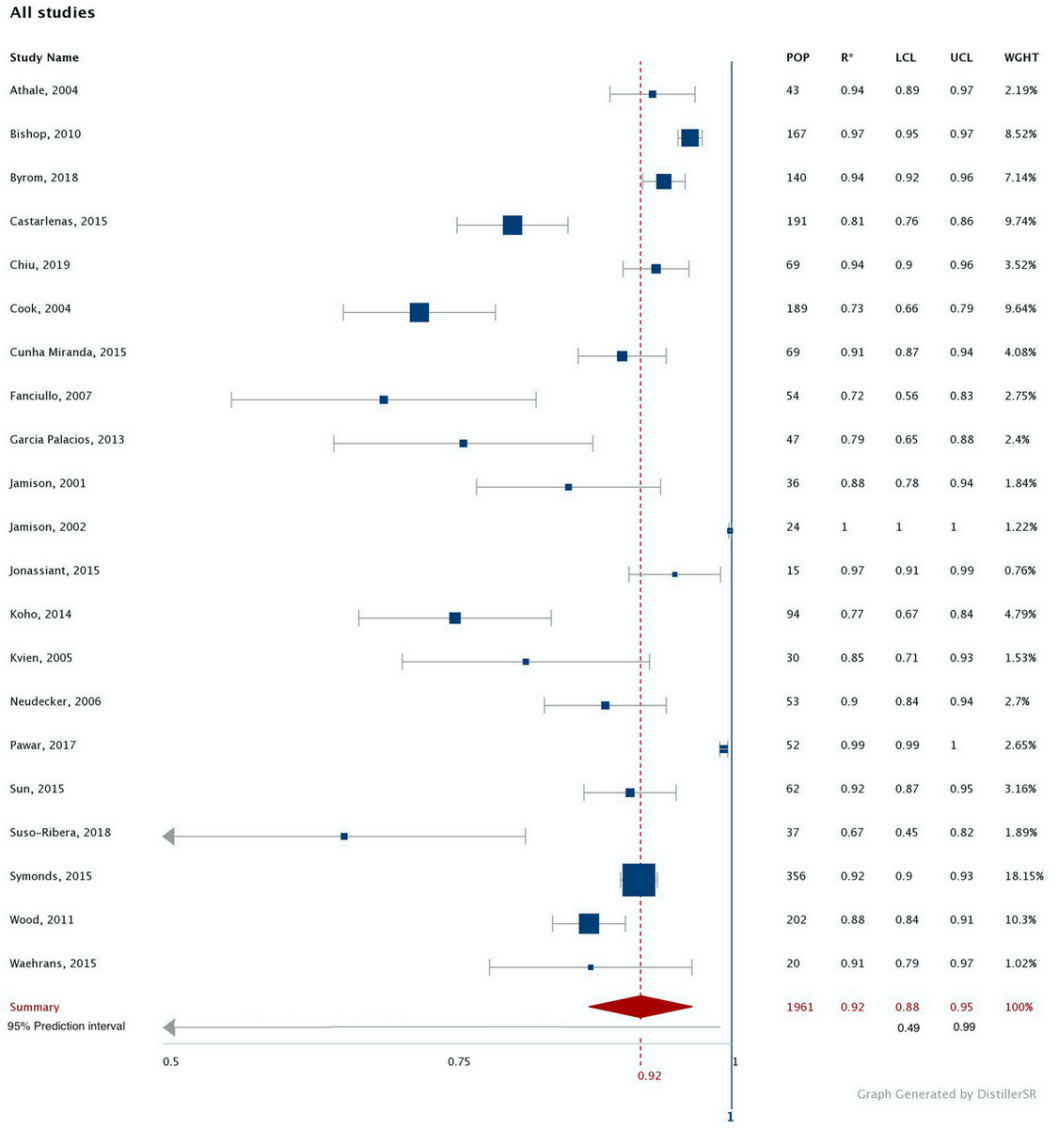
Summary correlation coefficient for pain intensity and interference data collected via electronic and conventional data capture methods (The I^2^ and *P* values for heterogeneity are 95% and <0.00001 respectively; the Z and *P* values for the overall effect are 14.4 and <0.00001 respectively; POP: population; R*: correlation coefficient; LCL: lower confidence interval limit; UCL: upper confidence interval limit; WGHT: weight).

A forest plot for correlations for score equivalence between data collection modalities is shown in Figure 3. The weighted summary correlation coefficient was 0.92 (95% CI 0.88-0.95, n=1961) and considerable heterogeneity (*I^2^*=95%) was observed across studies. Studies using ICC or weighted kappa produced summary correlations that were similar in magnitude to those using Pearson or Spearman rho correlations (ie, 0.91, 95% CI 0.90-0.92, n=1360, *I^2^*=95%; and 0.85, 95% CI 0.82-0.87, n=1159, *I^2^*=95%, respectively). One study met our predefined criterion for extreme effect size [43]. Removing this study from the analysis did not substantially decrease the heterogeneity (*I^2^*=94%), and the summary correlation was essentially unchanged at 0.90 (95% CI 0.86.0.93, n = 1937). Visual inspection of the funnel plot showed asymmetry, suggesting a possible publication bias (Multimedia Appendix 2).

Most studies used the same measure (n=16) versus a different measure (n=5) to assess pain via electronic and conventional modalities, and heterogeneity was high in both subgroups. The summary correlation was 0.93 in studies using the same measure (95% CI 0.89-0.96, n=1475, *I^2^*=96%, 95% prediction interval=0.45-0.99) and 0.86 in studies using different measures (95% CI 0.74-0.93, n=526, *I^2^*=90%, 95% prediction interval −0.01-0.99). In the case of data collection duration, 14 studies collected pain data from participants once and 7 collected data on multiple occasions. The summary correlation was 0.92 in studies that collected pain data once (95% CI 0.88-0.95, n=1678, *I^2^*=95%, 95% prediction interval 0.57-0.99) and 0.92 in studies that collected pain data from participants more than once (95% CI 0.75.0.98, n=283, *I^2^*=96%, 95% prediction interval −0.61-0.99). Heterogeneity remained high despite stratification by the duration of data collection.

### Data Completeness

Overall, 45% (24/53) studies reported the completeness of data collected via electronic or conventional methods (Table 3). All of these studies compared an electronic data capture modality to paper-based assessments with 8% (2/24) paper-based assessments being mailed to participants. The assessment of data completeness differed across studies and was largely defined as either the percentage of study participants not completing pain assessments or the percentage of missing or incomplete pain assessments. In total, 37% (9/24) studies reported superior data completeness in the electronic data capture group, 33% (8/24) studies reported superior data completeness in the conventional data capture group, 8% (2/24) studies reported mixed results, and 20% (5/24) studies did not conduct a direct comparison between data collection modalities, but reported a high data completeness using electronic data capture.

**Table 3:** Summary of study results related to data completeness.

Authors (year)	Electronic data collection modality	Conventional data collection modality	Definitions
Allena et al (2012) [25]	Complete records: 98%	Not reported	Defined as the percent of participants completing all assessments
Athale et al (2004) [26]	Missing data: 7/63 (11%)	Missing data: 16/63 (25%)	Defined as the percent of participants completing assessments
Bandarian-Balooch et al (2017) [27]	—^a^	Long-paper diaries had significantly higher missing data scores in data completion than the e-diaries and short-paper diaries (*P*<.05). The short-paper diary had significantly more missing data than the mobile phone groups (*P*<.05) but was not significantly different than the computer group.	Defined as the number of missing items irrespective of inaccurate completion
Bedson et al (2019) [28]	Recordings were made on 73.3% of days	Not reported	Defined as percentage of days on which participants recorded data
Bishop et al (2010) [29]	Missing data: 15 responses (0.004% of items)	Missing data: 3 responses (0.0007% of items)	Defined as the total number of missed assessment items across all participants
Christie et al (2014) [33]	Response rate: 97.9%	Not reported	Defined as the percent of possible text message–based pain assessments completed cross all participants
Gaertner et al (2004) [38]	Missing data: 8% of all daily assessments	Missing data: 0% (participants reported retrospectively completing assessments when they forgot to do so at the scheduled time)	Defined as the percent assessments not completed across all participants over 14 days
Garcia-Palacios et al (2013) [39]	Complete records: 18.2 (86.66%)	Complete records: 11.1 (52.95%; *P*<.01)	Defined as mean number of complete assessments across participants out of possible records
Heiberg et al (2007) [40]	Median value for missing daily data entries: 1 for both periods	Median value for missing daily data entries: 0 for both periods	Defined as median number of missing assessments over 21 days
Jamison et al (2001) [15]	Compliance with reporting: 89.9%	Compliance with reporting: 55.9%	Defined as percent of assessments completed each day for 1 year (365 days; electronic assessments) and percent of assessments completed for 7 days each month for 1 year (84 days; conventional assessment)
Junker et al (2008) [46]	Not reported	Noticeably more missing data on the conventional method when compared with the electronic pain assessment	Defined as number of missing items across each assessment
Khan et al (2019) [47]	Mean number of queries: 1.53 (2.70)	Mean (SD) number of queries: 0.90 (0.87)	Defined as concerns about a specific data point raised by the data manager or study coordinator relating to inappropriate or missing data
Marceau et al (2007) [52]	Complete records: 397/461 (86.1%)	Complete records: 583/583 (100%)	Defined as the number of assessments completed across all participants
Palermo et al (2004) [56]	Compliance: 83.3%	Compliance: 46.7% (*P*<.001)	Defined as the percent of assessments completed over the 7 days
Ritter et al (2004) [58]	Response rate: 87.5%	Response rate: 83.1% (*P*=.19)	Defined as percent of participants who completed assessments
Rolfson et al (2011) [59]	Response rate: 49%	Response rate: 92% (*P*<.01)	Defined as percent of participants who completed assessments
Stinson et al (2008 and 2014) [5,24]	Response rate: 78% and 73% for 2- and 3-week study protocols, respectively	Response rate: 93% in week 1 and 92% in week 2 (not reported for 3-week protocol)	Defined as 100% when 3 diary entries were completed for each of the 14 or 21 days of data collection
Stinson et al (2012) [63]	Missing data using Mobile phones: 5.26% (younger children), 3.42% (older children); missing data using computer: 0% (younger children), 0.14% (older children)	Missing data: 0% (younger children), 1.16%/77 (older children; *P*=.047)	Defined as the percent of assessment items not answered by participants
Stinson et al (2015) [7]	Response rate: 72.2% and 47.1% for 2- and 3-week study protocols, respectively	Not reported	Defined as 100% when participants completed 2 diary entries per day for 14 days
Stomberg et al (2012) [64]	Response rate on the day of surgery: 35%; response rate on days 2-4 postoperatively: 100%; response rate on days 5-6 postoperatively: 69%	Response rate on the day of surgery: 41%; response rate on days 2-4 postoperatively: 100%; not required to complete questionnaire on days 5-6	Defined as the percent of participants completing assessments
Stone et al (2003) [65]	Response rate 3 prompts per day: 93.5%; response rate 6 prompts per day: 93.9%; response rate 12 per day 95.5%	Response rate: 100.0%	Defined as the percent of participants completing assessments
Suso-Ribera et al (2018) [67]	Response rate: 75.7%	Not reported	Defined as the percent of completed assessments out of all possible assessments
VanDenKerkhof et al (2003) [70]	NRS^b^ score documentation rate: 100%	NRS score documentation rate: 90-97%	Defined as the percentage of time an NRS score was documented during a patient encounter
VanDenKerkhof et al (2004) [71]	Complete records pain scores: 64.7%; complete records nausea, pruritis and sedation side effects: 100%; complete records hypotension side effect: 20.6%	Complete records pain scores: 43.6% (*P*=.07); complete records nausea, pruritis and sedation side effects: 12.8-33.3% of paper assessments (*P*=<.001); complete records hypotension side effect: 5.1% (*P*=.07)	Percent of assessments where outcome was recorded

^a^N/A: not applicable.

^b^NRS: Numerical Rating Scale.

### Ease of Use

The ease of use of electronic and/or conventional pain data capture methods was reported in 45% (24/53) studies (Table 4). Ease was assessed subjectively using administered quantitative or qualitative surveys or verbal reports in all studies. Overall, electronic data collection modalities were considered easy to use by patients in pain or their care providers. In 91% (22/24) of the studies, the electronic modality was considered easy to use, easy to understand, or easy to review or report pain. In all, 29% (7/24) studies conducted inferential testing comparing ease between pain data capture modalities. Of these studies, 57% (4/7) showed that electronic versions were significantly easier to use, 14% (1/7) study showed that the paper version was significantly easier to use, and 28% (2/7) studies showed no significant differences between groups.

**Table 4:** Summary of study results related to ease of use.

Study (year)	Electronic data collection modality	Conventional data collection modality	Conclusion
Allena et al (2012) [25]	Easy to understand: mean 8.7/10; easy to use: mean 8.9/10	Easy to understand: mean 8.3/10; easy to use: mean 7.9/10	Electronic format significantly (*P*<.01) easier.
Athale et al (2004) [26]	9/19 (47%) rated computer as easier	5/19 (26%) rated paper as easier	Not reported
Bandarian-Balooch et al (2017) [27]	Ease of use (all electronic methods combined): mean 6.58/10	Ease of use: mean 6.17/10	The long-paper diary was rated as significantly (*P*<.02) less easy to use than the other diaries
Bedson et al (2019) [28]	100% reported easy to read	Not reported	Not reported
Bishop et al (2010) [29]	17 comments on easy completion	16 comments on easy completion	Not reported
Blum et al (2014) [30]	79% reported no difficulty with using electronic method	Not reported	Not reported
Cook et al 2004 [34]	39% of patients stated easier to understand and complete	24% of patients stated easier to understand and complete	Not reported
Freynhagen et al (2006) [37]	No issues with the use of the PDA^a^	Not reported	Not reported
Gaertner et al (2004) [38]	54% found more complicated	42% found more complicated	No significant difference between modalities
Garcia-Palacios et al (2013) [39]	15/40 (37%) rated easier to use	4/40 (10%) rated easier to use	Not reported
Jaatun et al (2014) [42]	Both physicians found electronic pain reports easier to read and evaluate than the paper maps.	Not reported	Not reported
Koho et al (2014) [49]	64/93 (69%) rated easy to complete, 10/93 (11%) rated difficult to complete	63/93 (68%) rated easy to complete, 10/93 (11%) rated difficult to complete	Not reported
MacKenzie et al (2011) [51]	54/63 (85.7%) rated easy to complete	Not reported	Not reported
Marceau et al (2007) [52]	32/36 (89%) rated easy to understand and use; 30/36 (83%) rated easy to record data	27/36 (75%) rated easy to understand and use; 3/36 (8%) rated easy to record data	No significant difference in ease of understanding and use. Significantly (*P*<.001) higher ease of recording data rating for electronic modality.
Marceau et al (2010) [53]	29/43 (67.4%) rated easy to use and understand	32/35 (91.4%) rated easy to use and understand	Significantly (*P*=.01) higher ease of use and understanding for paper modality.
Palermo et al (2004) [56]	15/18 (83%) rated easy or very easy to remember to fill out	8/15 (53%) rated easy or very easy to remember to fill out	No significant difference between modalities
Pawar et al (2017) [57]	70.58% rated as easy to use	Not reported	Not reported
Serif et al (2005) [62]	Some users, especially those with arthritis and/or poorer eyesight encountered difficulties in using the electronic modality, but ease of use was general consensus	Not reported	Not reported
Stinson et al (2008 and 2014) [5,24]	Majority found the electronic format easy to use	Not reported	Not reported
Stinson et al (2012) [63]	19/21 (91%) of parents the computer or paper to be easier to understand than the handheld device	Not reported	Significant difference (*P*=.03) in opinion of ease of use
Stinson et al (2015) [7]	94.6% and 91.7% of participants in the 2- and 3-week studies, respectively, found electronic diary interfered only minimally with activities	Not reported	Not reported
Stomberg et al (2012) [64]	Mean difficulty in using electronic modality: 1.31/10	No difficulties with use described	Not reported
Suso-Ribera et al (2018) [67]	100% of participants found the app extremely easy to use	Not reported	Not reported
Wæhrens et al (2015) [72]	Not reported	None found paper easier to use	Not reported

^a^PDA: personal digital assistant.

### Efficiency

In total, 30% (16/53) studies reported on the time to complete pain assessments (Table 5). In all, 44% (7/16) of these studies provided some evidence that pain assessments completed via the electronic modality were quick to complete; 19% (3/16) of these studies provided some evidence that conventional methods to assess pain were quicker; and 1 of 16 studies (6%) showed mixed results where differences in between-assessment modality completion times differed by participant group (eg, older children, parents, and younger children). In all, 25% (4/16) studies indicated that there were no differences in time to complete assessments across methods. Overall, in studies that directly measured the time to complete pain assessments [28,50,51,57,62,63,70,71], the difference in mean times to complete assessments was minimal (ie, <5.6 min).

**Table 5:** Summary of study results related to efficiency.

Study	Electronic data collection modality	Conventional data collection modality	Study author conclusions
Bedson et al (2019) [28]	Mean and max times to complete pain assessment: 2 and 5 min	Not reported	Not reported
Bishop et al (2010) [29]	19 comments on quick to complete	9 comments on quick to complete	Not reported
Blum et al (2014) [30]	70% completed pain assessment in under 5 min	88% completed pain assessment in under 5 min (questionnaire had fewer times than electronic modality)	Not reported
Gaertner et al (2004) [38]	No difference in time to complete pain assessments between groups (always less than 15 min/day)	—^a^	Not reported
Heiberg et al (2007) [40]	Time to complete the pain assessment similar between groups	—	Not reported
Kim et al (2016) [48]	68.7% responded that the time to complete pain assessments *positive* or *very positive*	Not reported	Significant relationship regarding participants evaluation of the time to complete electronic questionnaire *P*<.001
Kvien et al (2005) [50]	Mean (SD) time to complete pain assessment: 30.5 (16.0) min	Mean (SD) time to complete pain assessment: 24.9 (27.0) min	No significant difference between groups (*P*=.11)
MacKenzie et al (2011) [51]	Mean time to complete pain assessment: 25.0 min (range 5 to 80 min)	Mean time to complete pain assessment: 24.2 min (range 5 to 60 min)	Not reported
Pawar et al (2017) [57]	Mean time to complete pain assessment: 1.28 min (range 0.83-2.63 min)	Mean time to complete pain assessment: 3.7 min (range 2.42-5.23 min)	Not reported
Serif et al (2005) [62]	Mean time to complete pain assessment: 47 seconds	Mean time to complete pain assessment: 267 seconds	Not reported
Stinson et al (2008 and 2014) [5,24]	Most adolescents found the app quick to complete	Not reported	Not reported
Stinson et al (2012) [63]	Computer: mean (SD) time to complete pain assessment: 3.40 (1.53) min for older children, 4.00 (1.71) min for parents and 1.64 (1.50) min for younger children; Mobile phone: mean (SD) time to complete pain assessment: 5.90 (2.79) min for older children, 7.00 (4.08) min for parents and 1.82 (1.17) min for younger children	Mean (SD) time to complete pain assessment: 3.08 (1.66) min for older children, 2.28 (1.32) min for parents and 1.91 (1.81) min for younger children	Completion times significantly longer in electronic group for older children and parents (*P*=.001). No significant difference for younger children (*P*=.64) who completed a shorter assessment.
Stinson et al (2015) [7]	93.2% and 91.7% of participants in the 2- and 3-week studies, respectively, found electronic diary quick to complete	Not reported	Not reported
Stomberg et al (2012) [64]	Participants reported electronic modality not time consuming	Not reported	Not reported
VanDenKerkhof et al (2003) [70]	Median (IQR) time to complete pain assessment: 206 (70) seconds	Median (IQR) time to complete pain assessment: 153 (85) seconds	Completion time significantly longer time to complete using electronic modality (*P*<.001)
VanDenKerkhof et al (2004) [71]	Median (IQR) time to complete pain assessment 2.8 min	Median (IQR) time to complete pain assessment 2.7 min	No significant difference between groups (*P*=.74)

^a^N/A: not applicable.

### Acceptability

Data related to the comparative acceptability of each pain assessment modality were collected in 60% (32/53) studies [5,7,21,22,24-27,29,30,34,36,38-42,47-53,56,57,60,61,63,64,66,69,72]. Overall, electronic programs to assess pain are highly acceptable to patients. In total, 19 (83%) of the 23 studies [21,22,25,26,30,34,36,38-42,49-51,57,60,72,73] that directly surveyed patients reported that the electronic format was the preferred data collection method, compared with 1 of 23 studies (4%) [69], which reported that the conventional data collection method was preferable. This study indicated that age was related to patient preference, with younger patients (mean age 45 years) tending to prefer the internet and older patients (mean age 54 years), preferring the telephone-based data collection; 9% (2/23) studies reported discrepant results [66]. One of these studies reported that children aged <8 years favored the electronic assessment method, whereas the parents of these children and children aged 8 to 18 years had no preference. The other study reported that the preferred modality differed depending on the type of pain measurement instrument used. One study (4%) found no difference in participant satisfaction between electronic and conventional pain instruments [47]. Nine studies did not ask patients to specifically declare a preference for assessment modality but still reported high patient satisfaction with the electronic method [5,7,27,29,48,52,53,56,64,74].

## Discussion

### Principal Findings

This is the first systematic review and meta-analysis to compare electronic and conventional data collection methods for pain-related outcomes. The results from our review suggest strong correspondence in pain scores collected across electronic and conventional modalities as well as ease of use and acceptability for electronic data capture methods. Comparisons of data completeness and efficiency showed mixed results in terms of the superiority of electronic modalities over conventional methods. Overall, these results indicate that electronic data capture is a viable means to assess pain and has the potential to overcome many of the known limitations associated with conventional methods.

The capacity to obtain equivalently scored data from patients across electronic and conventional data capture modalities is paramount to the use of more novel collection methods in clinical and research settings. Studies included in this review (ie, in 82% of cases) commonly reported on the correspondence of pain scores between assessments. Regardless of whether the data analyses were qualitative or quantitative, the general consensus across studies was that pain was reported equivalently across assessment modalities. The meta-analysis of correlations between scores reported electronically and conventionally resulted in a summary coefficient of 0.92, indicating high correspondence. The summary coefficients produced by studies reporting ICC or weighted kappa and studies reporting Pearson or Spearman rho coefficients were not different from the overall summary score, suggesting negligible change in patient-reported scores across modalities. These findings agree with those of a meta-analysis published in 2008 that evaluated the equivalence of scores for patient-reported outcomes (not specifically pain) completed using PDA, computer, or tablet and paper-based methods and that showed a summary correlation of 0.90 [9]. Together, these reviews suggest that score equivalence between electronic and conventional data capture methods is a robust finding across patient-reported outcomes.

Despite our use of random effects models, we observed substantial heterogeneity across studies included in the meta-analysis that was not accounted for by the single study that met our criterion for extreme effect size, sensitivity analyses by correlation type, the similarity of pain assessment measure used in each modality, or duration of data collection. Studies varied in terms of study design, participant group, type of electronic and conventional data collection method, and pain measurement instrument—the heterogeneity may be explained by these differences in methodology. For instance, the type of electronic device used to collect pain data varied across studies, meaning that aspects of the device such as interface design, user familiarity, and screen size could each have contributed to our heterogeneous results [11]. The included studies also varied in terms of the type of pain intensity scale or pain interference instrument used (eg, NRS, VAS, etc). Although good congruence in patient self-report across instruments has been shown [75], and that the transfer of the assessment instrument to the electronic format generally appeared to be in good faith, as reported previously, differences in pain ratings across instruments are possible [76]. Irrespective of the observed heterogeneity, the correlation coefficients were strong across all studies with no reported coefficients less than 0.64, suggesting that heterogeneity should not temper the meta-analysis conclusion.

The collection of high-quality and complete patient-reported data is of utmost importance to clinicians, researchers, and study sponsors [12]. Data completeness was a commonly reported comparison outcome across data collection methods in the included studies. The results regarding the superiority of data completeness were mixed. However, the electronic method was most often associated with more complete data being collected. Ultimately, methodological and logistical issues related to paper-based data collection methods may support the use of electronic data capture. For instance, research has shown that the completeness and accuracy of pain data collected via paper methods is adversely impacted by patients back-filling diaries and, therefore, introducing recall bias into datasets (a behavior that can be rendered impossible using electronic methods) [8]. In addition, the capacity to efficiently and cost-effectively develop large databases for clinical and research purposes may be improved with electronic data capture. For instance, one of the studies included in this review [47] showed that over 4-fold more research assistant time was required to manage postoperative pain data collected using conventional means compared with electronic data. This finding suggests that cost savings may result from the use of electronic pain assessments in research, and this savings might be pronounced at scale. Furthermore, the likelihood of inaccurate or missing data in these databases resulting from human input error is reduced in the case of electronic entry [77].

Almost all studies that assessed ease indicated, in some manner, that electronic methods were easy to use, easy to understand, or easy to review or report pain. The time difference required to complete pain assessments via each data collection method was minimal, and the majority of studies showed that the electronic method required equal or less time to complete than conventional methods. The methodological advantages of electronic data capture include high-density sampling in all environments. Evidence of ease of use and efficiency in electronic data capture is useful to researchers and clinicians considering leveraging these utilities to collect repeated ecologically relevant pain assessments [78].

Electronic data capture was also shown to be a highly acceptable method for pain assessment and was more likely to be the method of choice for reporting by patients. These findings agree with those of previous studies comparing electronic and conventional methods [10]. Given the heterogeneity of electronic pain data capture methods, participant populations, and sampling densities of included studies, our results suggest acceptability across a range of data collection contexts. This result is meaningful as the acceptability of an intervention has been linked to adoption, especially in relation to long-term sustainability [79].

### Limitations

Some included studies did not administer the same pain measurement instrument or use the same sampling schedule via electronic and conventional methods, making it difficult to directly compare results across modalities. Owing to variations in study design and the fact that our outcomes of interest were often times not the main objective of our included studies, we did not perform an assessment of quality for included studies; instead, we elected to include all identified studies in our review. Our results and conclusions are, therefore, the product of studies that may have included significant methodological weaknesses. In addition, as is an issue with all systematic reviews, we are constrained by possible publication bias, which was suggested by the funnel plot inspection of our quantitative synthesis data. However, given the objectives of the studies we included, we believe that the likelihood of a *file-drawer effect* is low. Finally, we included studies conducted in controlled (eg, research and health care institutions) and uncontrolled (eg, participant home) environments. We are, therefore, limited in our ability to make more definitive conclusions about our outcomes as they pertain to ecologically relevant data collection, which is considered a major methodological advantage of the electronic method.

### Conclusions

Overall, this review demonstrates that electronic pain-related data capture methods are comparable with conventional methods in terms of score equivalence, data completeness, ease, efficiency, and acceptability. Specifically, pain-related outcome scores reported across methods were congruent in terms of score correlations and mean or median differences between scores. Data completeness, ease of use, efficiency, and acceptability outcomes were also comparable or superior using electronic data capture. Our results suggest that electronic methods are a feasible means to collect pain data, and the use of these methods is likely to increase with the ubiquitous use of mobile phones outside of the clinical or research setting. However, a critical caveat to this conclusion relates to the validation of pain instruments that are implemented electronically. To ensure the collection of accurate data, rigorous methods should be used to establish the sound psychometric properties of electronic pain measurement instruments. Validation of electronic methods will facilitate the capture of pain data in clinical settings but will also support its use in data collection for interventional research, an area that has largely not been explored to date [6].

## Appendix

Multimedia Appendix 1 Sample search strategy.

Multimedia Appendix 2 Funnel plot of 21 studies presenting correlations for score equivalence between electronic and conventional pain data collection modalities.

## Abbreviations

ICC: intraclass correlation coefficient
NRS: Numerical Rating Scale
PDA: personal digital assistant
VAS: Visual Analog Scale
