# Supplementary material for: Electronic Data Capture Versus Conventional Data Collection Methods in Clinical Pain Studies: Systematic Review and Meta-Analysis
Source: J Med Internet Res. 2020 Jun 16;22(6):e16480. doi: 10.2196/16480 (PMC7351264; doi:10.2196/16480)
Supplement: Multimedia Appendix 1 [file jmir_v22i6e16480_app1.docx]

1. exp Pain/

2. Pain Measurement/

3. Pain Threshold/

4. exp Pain Perception/

5. Pain.mp.

6. pain reporting.mp.

7. 1 or 2 or 3 or 4 or 5 or 6

8. Electronics/

9. exp Cellular Phone/

10. exp Computers, Handheld/ or exp Wireless Technology/ or exp Internet/ or exp Computer Communication Networks/

11. exp Mobile Applications/ or smartphone.mp.

12. personal digital assistant.mp.

13. computers/ or computers, handheld/

14. 8 or 9 or 10 or 11 or 12 or 13

15. 7 and 14

16. exp Randomized Controlled Trials as Topic/ or exp Randomized Controlled Trial/ or randomized.mp.

17. exp Multicenter Study/ or exp Observational Study/

18. Humans/

19. exp Adult/ or exp Prospective Studies/ or prospective.mp.

20. 16 or 17 or 18 or 19

21. 15 and 20

22. limit 21 to yr="2018 -Current"

23. limit 22 to english
