# Supplementary figures and images for: Electronic Data Capture Versus Conventional Data Collection Methods in Clinical Pain Studies: Systematic Review and Meta-Analysis
Source: J Med Internet Res. 2020 Jun 16;22(6):e16480. doi: 10.2196/16480 (PMC7351264; doi:10.2196/16480)

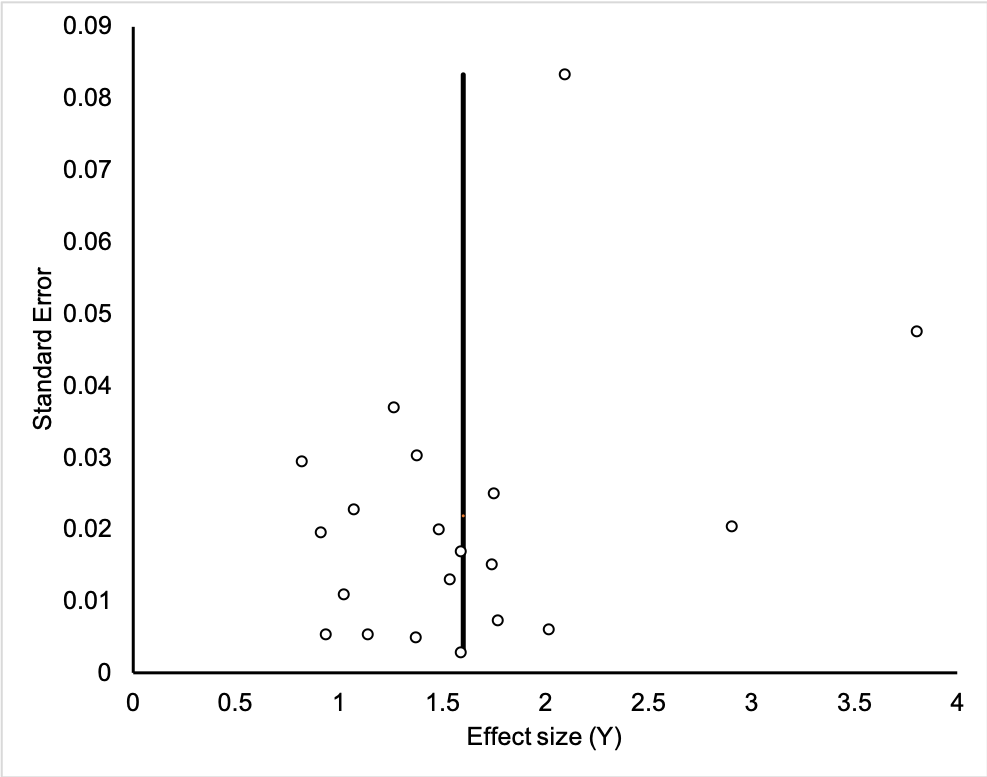

Supplement: Multimedia Appendix 2 [file jmir_v22i6e16480_app2.png]
